# Pristimerin induces apoptosis in imatinib-resistant chronic myelogenous leukemia cells harboring T315I mutation by blocking NF-κB signaling and depleting Bcr-Abl

**DOI:** 10.1186/1476-4598-9-112

**Published:** 2010-05-19

**Authors:** Zhongzheng Lu, Yanli Jin, Chun Chen, Juan Li, Qi Cao, Jingxuan Pan

**Affiliations:** 1 Department of Pathophysiology, Zhongshan School of Medicine, Sun Yat-sen University, Guangzhou, PR China; 2 Department of Pediatrics, Sun Yat-sen memorial Hospital, Sun Yat-sen University, Guangzhou, PR China; 3 Department of Hematology, The First Affiliated Hospital, Sun Yat-sen University, Guangzhou, PR China

## Abstract

**Background:**

Chronic myelogenous leukemia (CML) is characterized by the chimeric tyrosine kinase Bcr-Abl. Bcr-Abl-T315I is the notorious point mutation that causes resistance to imatinib and the second generation tyrosine kinase inhibitors, leading to poor prognosis. CML blasts have constitutive p65 (RelA NF-κB) transcriptional activity, and NF-κB may be a potential target for molecular therapies in CML that may also be effective against CML cells with Bcr-Abl-T315I.

**Results:**

In this report, we discovered that pristimerin, a quinonemethide triterpenoid isolated from Celastraceae and Hippocrateaceae, inhibited growth and induced apoptosis in CML cells, including the cells harboring Bcr-Abl-T315I mutation. Additionally, pristimerin inhibited the growth of imatinib-resistant Bcr-Abl-T315I xenografts in nude mice. Pristimerin blocked the TNFα-induced IκBα phosphorylation, translocation of p65, and expression of NF-κB-regulated genes. Pristimerin inhibited two steps in NF-κB signaling: TAK1→IKK and IKK→IκBα. Pristimerin potently inhibited two pairs of CML cell lines (KBM5 versus KBM5-T315I, 32D-Bcr-Abl versus 32D-Bcr-Abl-T315I) and primary cells from a CML patient with acquired resistance to imatinib. The mRNA and protein levels of Bcr-Abl in imatinib-sensitive (KBM5) or imatinib-resistant (KBM5-T315I) CML cells were reduced after pristimerin treatment. Further, inactivation of Bcr-Abl by imatinib pretreatment did not abrogate the TNFα-induced NF-κB activation while silencing p65 by siRNA did not affect the levels of Bcr-Abl, both results together indicating that NF-κB inactivation and Bcr-Abl inhibition may be parallel independent pathways.

**Conclusion:**

To our knowledge, this is the first report to show that pristimerin is effective *in vitro *and *in vivo *against CML cells, including those with the T315I mutation. The mechanisms may involve inhibition of NF-κB and Bcr-Abl. We concluded that pristimerin could be a lead compound for further drug development to overcome imatinib resistance in CML patients.

## Background

Chronic myelogenous leukemia (CML) is a myeloproliferative disorder characterized by the presence of Bcr-Abl which is formed by a reciprocal chromosomal translocation t(9,22)(q34;q11) [1,2]. Bcr-Abl is essential for malignant transformation [3], and triggers several cellular signaling pathways (e.g., CrkL, STAT5, PI3K/AKT) to regulate cell proliferation, differentiation, migration, survival and DNA repair [4]. Targeting Bcr-Abl has therefore been an important strategy for CML treatment [5]. Imatinib (STI571, Gleevec, Norvartis) effectively inhibits tyrosine kinase activity by occupying the adenosine triphosphate (ATP)-binding pocket of Bcr-Abl, thus abrogating subsequent signal transduction [6]. Of newly diagnosed patients with chronic-phase CML, 82% showed complete cytogenetic response on treatment with imatinib over a median follow-up of 54 months [7]. However, resistance to imatinib develops over time and is an emerging problem for CML patients. Most cases of acquired clinical resistance are due to mutations in the kinase domain of Bcr-Abl. To overcome the acquired resistance, second generation tyrosine kinase inhibitors (e.g., nilotinib, dasatinib and INNO-406) have been developed and are effective against all but the T315I mutation which accounts for approximately 20% of acquired resistance cases [8-11]. Hence, novel strategies or compounds that can inhibit/kill CML cells carrying Bcr-Abl-T315I are needed.

Abnormal constitutive NF-κB activation is widely found in diverse types of hematopoietic malignancies (e.g. CML [12], acute myeloid leukemia [13], Hodgkin's disease [14]) as well as solid tumors. In particular, the constitutive activation of NF-κB exists selectively in leukemia stem cells but not in normal hematopoietic stem cells [15]. Therefore, NF-κB may be a potential therapeutic target for the selective eradication of leukemia stem cells. CML blasts have constitutive p65 (RelA NF-κB) transcriptional activity, and NF-κB may be a potential target for molecular therapies in CML [16]. Indeed, pharmacological inhibition of NF-κB was effective in killing CML cells [17].

Pristimerin is a quinonemethide triterpenoid compound isolated from Celastraceae and Hippocrateaceae. Pristimerin has shown antimicrobial, anti-inflammatory, antiperoxidation activities [18] as well as antitumor effects in various types of human cancers [19-21]. Although pristimerin is known to be a potent inhibitor of nuclear factor κB (NF-κB)-mediated transcription [20], the detail mechanism by which pristimerin inhibits activation of NF-κB is unknown. In our continuing search for effective compounds to kill CML cells carrying T315I-Bcr-Abl [22,23], we tested this NF-κB inhibitor (pristimerin) against CML cells including those carrying T315I-Bcr-Abl, and elaborated the mechanistic detail of the inhibition of NF-κB by pristimerin. We extended our work to translational studies evaluating the *in vivo *efficacy of pristimerin against imatinib-resistant cells. We also found that downregulating the levels of Bcr-Abl may also be a relevant antineoplastic mechanism of pristimerin in malignant cells carrying Bcr-Abl.

## Methods

### Chemicals and antibodies

**Figure 1:**
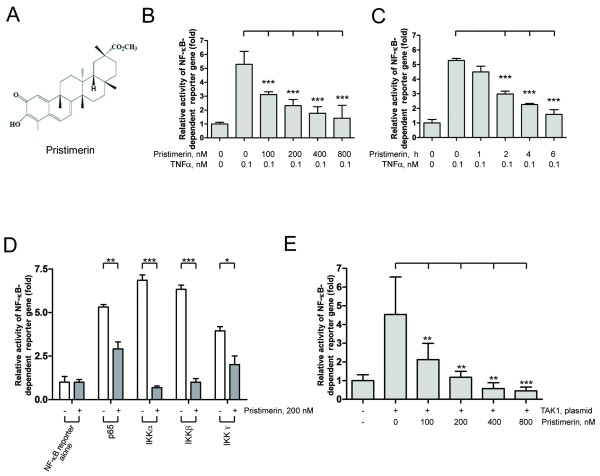
**Pristimerin abrogated TNF-induced NF-κB activation at TAK1 and IKK steps**. (A) Structure of pristimerin. (B and C) Pristimerin inhibited TNFα-induced NF-κB-dependent reporter gene expression. U2OS cells were cotransfected with 60 ng NF-κB-TATA-Luc reporter plasmid and 10 ng Renilla luciferase reporter plasmid. 24 hours later, cells were treated with different concentrations of pristimerin (B) or a fixed concentration (200 nM) for various durations (C), then TNFα (0.1 nM) for 10 minutes; cell supernatants were prepared, and luciferase intensity was measured. The levels of firefly luciferase activity were normalized to Renilla luciferase activity. Results were expressed as fold change ± SE of at least 3 independent experiments. ***, *P *< 0.0001, one-way ANOVA, post hoc comparisons, Tukey's test. Columns, mean; error bars, SE. (D) Pristimerin abrogated IKK-induced NF-κB activation. U2OS cells were cotransfected with the indicated plasmids (p65, IKKα, IKKβ, IKKγ) along with NF-κB-TATA-Luc reporter plasmid. 24 hours later, cells were treated with 200 nM pristimerin for 6 hours. The expression of Renilla luciferase activity normalized transfection efficiency. *, *P *< 0.05; **, *P *< 0.01; ***, *P *< 0.0001, one-way ANOVA, post hoc comparisons, Tukey's test. Columns, mean; error bars, SE. (E) Pristimerin diminished TAK1-induced NF-κB activation. U2OS cells were cotransfected with the TAK1-expressing plasmid and NF-κB-TATA-Luc reporter plasmid. 24 hours later, cells were treated with increasing concentrations of pristimerin for 6 hours. Luciferase activity was assayed as described above. **, *P *< 0.01; ***, *P *< 0.0001, one-way ANOVA, post hoc comparisons, Tukey's test. Columns, mean; error bars, SE.

Pristimerin (Figure 1A, purity > 98%, HPLC) was obtained from Paypaytech Inc. (Shenzhen, China). Pristimerin was dissolved in DMSO (Sigma, Shanghai) at a stock concentration of 10 mM, and stored at -20°C. Imatinib (STI571) was purchased from Alexis Biochemicals (Plymouth Meeting, PA). Annexin V-FITC, and mouse monoclonal antibody against actin were from Sigma-Aldrich (Sigma, Shanghai). MG-132 was from Calbiochem (San Diego, CA). Recombinant human TNFα was from Peprotech (Rocky Hill, NJ). Antibodies against p65, IκBα, c-Abl (C-19), proliferating cell nuclear antigen (PCNA), IKKα, cyclin D1 (C-20), apoptosis-inducing factor (AIF, H300), Bax, Bcl-X_L_, and Mcl-1 (S-19) were from Santa Cruz Biotechnology (Santa Cruz, CA). Mouse antibodies against poly(adenosine diphosphate [ADP]-ribose) polymerase (PARP, clone 4C10-5), caspase-3, and active caspase-3 (CM1), XIAP, cyclooxygenase-2 (Cox-2), and cytochrome c (clone 6H2.B4) were from BD Biosciences (San Jose, CA). Antibodies against phospho-IKKα (Ser180)/IKKβ (Ser181), phospho-IκBα at Ser32, phospho-p65 at Ser536, phospho-c-Abl at Y245, phospho-Erk1/2 (T202/Y204), Erk1/2, phospho-AKT at Ser473 and AKT were from Cell Signaling Technology (Beverly, MA). Antibodies against phospho-STAT5A/B (Y694/Y699) (clone 8-5-2), STAT5A, and Bcl-2 were from Upstate Technology (Lake Placid, NY). Anti-phospho-RNA polymerase II (S5) was from Bethyl Laboratories (Montgomery, TX); Anti-survivin was from Novus Biol (Littleton, CO). Anti-mouse immunoglobulin G and anti-rabbit immunoglobulin G horseradish peroxidase-conjugated secondary antibodies were from Pierce Biotechnology (Rockford, IL).

### Cell culture

Imatinib-sensitive KBM5 cells bearing 210 kDa wild-type Bcr-Abl, were grown in Iscove's modified Dulbecco's medium (Invitrogen, Guangzhou, China) supplemented with 10% heat-inactivated fetal calf serum (FCS), as described previously [22,23]. Imatinib-resistant KBM5-T315I cells bearing T315I mutation in Bcr-Abl were routinely maintained in the same medium but with 1.0 μM imatinib, which was removed before experiments with a wash-out period of 2-3 days. KBM5 and KBM5-T315I had different sensitivities to imatinib; IC_50 _values were 0.28-0.53 and 5.04-5.40 μM, respectively [22,23]. The 32D myeloid cells stably expressing either 210 kDa wild-type Bcr-Abl (32D-Bcr-Abl) or T315I Bcr-Abl (32D-T315I) were established and maintained in RPMI 1640 with 10% FCS as described previously [23]. K562 cell was grown in RPMI 1640 with 10% FCS. U2OS cells were cultured in DMEM with 10% FCS. Cells in logarithmic phase were used in all experiments starting with 2.0 × 10^5 ^cells/mL.

### Primary cells from CML patients

Peripheral blood samples were obtained from 7 patients with leukemia [5 CML, 1 JMML (juvenile myelomonocytic leukemia), 1 ALL (acute lymphoid leukemia), Additional file 1, Table S1] and 4 healthy adult donors in Sun Yat-sen Memorial Hospital of Sun Yat-sen University, and The First Affiliated Hospital of Sun Yat-sen University after informed consent according to the institutional guidelines and the Declaration of Helsinki principles. Mononuclear cells were isolated by Histopaque gradient centrifugation (density 1.077 g/mL; Sigma-Aldrich, Shanghai, China). Contaminating red cells were lysed in 0.8% ammonium chloride solution for 10 minutes. After washing with PBS, cells were suspended in RPMI 1640 supplemented with 10% FCS.

### Luciferase assay

U2OS cells (2 × 10^5^) were transfected with reporter plasmids encoding NF-κB-TATA-luc (60 ng) and pEF*Renilla*-luc (10 ng) or together with plasmids encoding the desired genes by use of LipofectAMINE 2000 (Invitrogen). After 24 hours, cells were left untreated or exposed to pristimerin for 6 hours. The cell supernatants were prepared immediately after stimulation with TNFα (0.1 nM), and luciferase activities were measured with use of dual-luciferase assay kits (Promega, Madison, WI) as described previously [24]. NF-κB activities were determined by normalization of NF-κB-dependent firefly luciferase to Renilla luciferase activity. The pNF-κB-Luc plasmid was from Stratagene (La Jolla, CA). The plasmids pCMV5-IKKα, pCMV5-IKKβ, pCMV5-IKKγ, pCMV5-p65, and pCMV5-myc-TAK1 used for co-transfection experiments were described previously [24].

### Electrophoretic mobility shift assay (EMSA)

**Figure 2:**
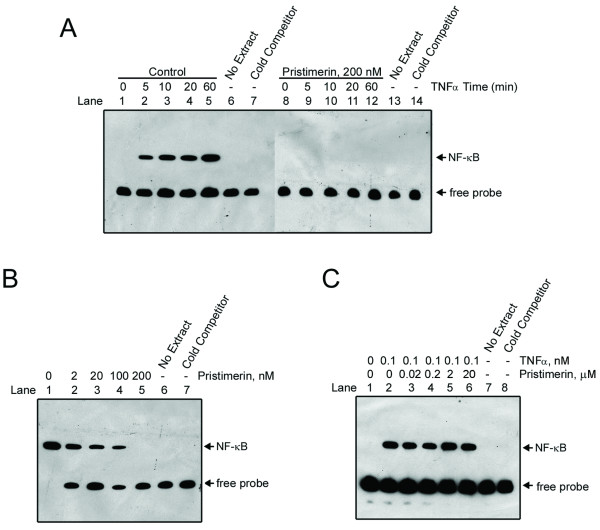
**Pristimerin inhibited DNA binding of NF-κB to DNA**. (A) KBM5 cells were preincubated in the absence or presence of 200 nM pristimerin for 6 hours; TNFα was added at different times, and the nuclear extracts were then assayed for NF-κB activation by EMSA. Cold competitor: the labeled NF-κB oligonuceotide was competed with an excess (200 fold) of unlabeled probe. (B) KBM5 cells were pretreated with or without escalating concentrations of pristimerin for 6 hours; TNFα (0.1 nM) was added for 30 minutes. EMSA was used to detect the activation of NF-κB. Results are representative of 3 independent experiments. (C) Pristimerin did not directly interfere with NF-κB complex formation. Nuclear extracts from KBM5 cells treated with or without 0.1 nM TNFα for 30 minutes, were reacted in the absence or presence of increasing concentrations of pristimerin, then NF-κB activation was assayed by EMSA.

EMSA involved use of the LightShift Chemiluminescent EMSA kit (Pierce Biotechnology, Shanghai) according to the manufacturer's instructions [24]. The oligonucleotides for NF-κB were from Promega (Shanghai, China): forward, 5-AGT TGA GGG GAC TTT CCC AGG C-3; reverse, 5-G CCT GGG AAA GTC CCC TCA ACT-3. The binding specificity was examined by competition with a 200-fold excess of the unlabeled oligonucleotide probe (cold competitor, Figure 2A, lanes 7 and 14).

### Preparation of whole cell lysates and cytosolic fraction

Control or drug-treated cells were pelleted by centrifugation and rinsed with PBS. The whole lysates were then prepared with RIPA buffer [22,25]. The cytosolic fraction was prepared with digitonin extraction buffer to detect the level of cytochrome c and AIF in the cytosol, as described previously [22,25].

### Preparation of cytoplasmic and nuclear fractions

TNFα- and/or pristimerin-treated cells were pelleted by centrifugation and rinsed with PBS. Pellets were then resuspended in 200 μl ice-cold lysis buffer (10 mM Hepes pH 7.9, 10 mM KCl, 0.1 mM EDTA, 0.4% NP 40 with 1 mM DTT, 0.5 mM PMSF, 1 mM sodium orthovanadate and Complete Protease Inhibitor Mix) by pipetting up and down (without bubbling) about 10 times [24,26]. After incubation on ice for 10 minutes, the lysates were centrifuged at 10,000 g. The supernatants were transferred to fresh tubes and referred to as cytoplasmic extracts. After washing with the lysis buffer, the pellets were vigorously resuspended in nuclear protein extraction buffer with inhibitors (20 mM Hepes, pH 7.9, 0.4 M NaCl, 1 mM EDTA with 1 mM DTT, 0.5 mM PMSF, 0.2 mM sodium orthovanadate and Complete Protease Inhibitor Mix) and centrifuged for 10 minutes at 14,000 g speed at 4°C. The resultant supernatants were kept as nuclear fractions [24,26].

### Immunofluorescence staining

After being preincubated with or without pristimerin for 6 hours, KBM5 cells were stimulated with TNFα. The cells were harvested and cytospinned onto glass slides. After fixation, and permeabilization, immunofluorescence staining was performed as described previously [27].

### Reverse transcription quantitative real-time PCR (RT-qPCR)

DNA-free total RNA was prepared with RNeasy mini-columns, using the on-column DNAase digestion step (Qiagen, Valencia, CA) and reverse transcribed into cDNA following the instruction of Invitrogen SuperScript III First-Stand Kit. Quantitative real-time PCR was performed with Roche LightCycler 480 according to the manufacture's recommended protocol. Optimal reaction conditions for amplification of both Bcr-Abl and 18S were as follows: 40 cycles of a two-step PCR (95°C for 15 s, 60°C for 20 s) after initial denaturation (95°C for 30s) with 5 μl of cDNA reaction by TaKaRa SYBR Premix Ex Taq Kit. The PCR-primers was as follow: Bcr-Abl - sense: 5'-TCCACTCAGCCACTGGATTTAA-3', antisense:

5'-TGAGGCTCAAAGTCAGATGCTACT-3'; Sirt1 - sense:

5'-GAGTGGCAAAGGAGCAGATT-3', antisense:

5'-ATGTAACGATTTGGTGGCAA-3'; 18S - sense:

5'-AAACGGCTACCACATCCAAG-3', antisense:

5'-CCTCCAATGGATCCTCGTTA-3'. The mRNA expression of Bcr-Abl or Sirt1 was normalized as described previously [28]. The results of three independent experiments are reported.

### Small interfering RNA transfection

Small interfering RNA (siRNA) duplexes against p65 were from Cell Signaling Technology (Beverly, MA); ON-TARGETplus SMARTpoolsiRNA duplexes against human PDGFRa or ON-TARGETplus Non-Targeting Pool siRNA control were from Dharmacon RNA Tech. (Lafayette, CO) [27]. Transfection of either anti-p65, PDGFRα or the control synthesized siRNA duplexes into K562 cells was performed with the Cell Line Nucleofector Kit T (Amaxa, Gaithersburg, MD) and program O-17 according to the manufacturer's instruction [27]. Twenty-four hours after siRNA transfection, the cells were analyzed with Western blotting.

### Cell viability assay

Cell viability was determined by MTS assay (CellTiter 96 Aqueous One Solution Cell Proliferation assay; Promega, Madison, WI) [22,24]. An amount of 100 μl cells (2.0 × 10^5^/mL) were seeded in 96-well plates and incubated with various concentrations of pristimerin for 72 hours. Four hours before culture termination, 20 μL of MTS solution was added to each well. Absorbance was read on a 96-well plate reader at a wavelength of 490 nm. Control cells received DMSO (< 0.1%) containing medium. The drug concentration resulting in 50% inhibition of cell growth (IC_50_) was determined.

### Soft agar clonogenic assay

To evaluate anchorage-independent growth of tumor cells, CML cells (2 × 10^5^/ml) were treated with increasing concentrations of pristimerin or diluent (DMSO, control) for 24 hours, then washed with PBS; the same amounts of viable cells (as determined by trypan blue exclusion assay and a hemocytometer) were subsequently seeded in 6-well plates in Iscove's medium containing 0.3% agar and 20% FCS in the absence of drug treatment. After incubation for 10 ∼ 14 days at 37°C, colonies composed of more than 50 cells were counted using an inverted phase-contrast microscope, as described previously [22,24].

### Tumor xenograft experiments

Male *nu/nu *BALB/c mice were bred at the animal facility of Sun Yat-sen University. The mice were housed in barrier facilities with a 12-h light dark cycle, with food and water available *ad libitum*. 3 × 10^7 ^of KBM5-T315I cells were inoculated subcutaneously on the flanks of 4- to 6-week-old male nude mice. Tumors were measured every other day with use of calipers. Tumor volumes were calculated by the following formula: *a*^2 ^× *b *× 0.4, where *a *is the smallest diameter and *b *is the diameter perpendicular to *a*. Pristimerin was dissolved in DMSO and diluted with vehicle [30% Cremophor EL/ethanol (4:1), 70% PBS]. Mice in the control group received the same amount of vehicle treatment. The body weight, feeding behavior and motor activity of each animal were monitored as indicators of general health. The animals were then euthanized, and tumor xenografts were immediately removed, weighed, stored and fixed. All animal studies were conducted with the approval of the Sun Yat-sen University Institutional Animal Care and Use Committee.

### Immunohistochemical staining

Formalin-fixed xenografts were embedded in paraffin and sectioned according to standard techniques. Tumor xenograft sections (4 μm) were immunostained using the MaxVision kit (Maixin Biol, Fuzhou, China) according to the manufacturer's instructions [22,23].

### Flow cytometry analysis

#### Cell cycle analysis

Cells treated and control were harvested, washed with PBS, and fixed with 66% ethanol over night. Cells were centrifuged and washed with PBS, then stained with 50 μg/mL propidium iodide and 2.5 μg/mL RNase in PBS solution for 30 minutes at room temperature. DNA content was analyzed by flow cytometry at the emission wavelength of 488 nm [22].

#### Apoptosis measurement

Apoptosis was measured by flow cytometry using Annexin V/Propidium iodide (PI) double staining. Cells were cultured in the presence of indicated concentrations of pristimerin for 24 hours, harvested and washed, and incubated in binding buffer (Annexin V Binding Buffer, BD Pharmingen) with 0.3% Annexin V-FITC for 15 minutes at room temperature. The cells were washed and resupended in binding buffer. Propidium iodide was added just before flow cytometric analysis [22,24].

### Statistical analysis

GraphPad Prism 4.0 software (GraphPad Software, San Diego, CA) was used for statistical analysis. Comparisons between 2 groups involved two-sided Student's *t* test, and comparisons among multiple groups involved one-way ANOVA with post-hoc intergroup comparisons using Tukey test. *P *< 0.05 was considered statistically significant.

## Results

### Pristimerin inhibits TNF-induced NF-κB-dependent reporter gene transcription

We first examined whether pristimerin affected the TNFα-induced NF-κB-dependent reporter gene transcription. One day after cotransfection with pNF-κB-TATA-Luc and pEFR*Renilla*-Luc, U2OS cells were exposed to pristimerin at increasing concentrations for 6 hours or a fixed concentration (200 nM) for different durations. Prior to the termination of culture, TNFα was added for 10 minutes. The luciferase activity detected was increased by TNFα (Figure 1B); but pristimerin inhibited the TNFα-induced NF-κB reporter activity in a dose- and time-dependent manner (Figure 1B and 1C).

### Pristimerin inhibits NF-κB activation induced by p65, IKKα, IKKβ, IKKγ and TAK1

In the canonical NF-κB activation pathway, TAK1 and IKK are the major upstream regulators of IκBα. To determine the steps at which pristimerin acted, U2OS cells were cotransfected with plasmids to express IKKα, IKKβ or IKKγ, along with an NF-κB-TATA-Luc reporter plasmid. The luciferase activity of NF-κB-TATA-Luc reporter was significantly increased when cotransfected with p65, IKKα, IKKβ, or IKKγ constructs (Figure 1D) compared with transfection with reporter alone. However, addition of pristimerin significantly inhibited the NF-κB transcriptional activity (Figure 1D). Therefore, pristimerin could block NF-κB activation induced by IKK overexpression.

Because TAK1 is critical upstream regulator of IKK [29], we assessed the effect of pristimerin on cotransfection of a TAK1 construct along with NF-κB-TATA-Luc reporter plasmid. TAK1 significantly elevated NF-κB reporter luciferase activity (Figure 1E), and pristimerin significantly blocked TAK1-induced NF-κB activation.

### Pristimerin inhibits DNA binding of NF-κB in intact cells but does not directly interfere with binding of NF-κB to DNA in a purified nuclear extract

We next examined whether pristimerin interfered with the binding of NF-κB to DNA by EMSA. KBM5 cells were preincubated with or without 200 nM pristimerin for 6 hours; TNFα was added for the indicated times, then nuclear extracts were assayed for NF-κB DNA binding activity by EMSA with a probe representing an NF-κB-binding site. After stimulation with TNFα, the levels of the NF-κB-DNA complex were steadily increased over time in the absence of pristimerin (Figure 2A, lanes 2-5 versus lane 1). With the same durations of stimulation with TNFα, NF-κB-DNA complex were not formed in the presence of 200 nM pristimerin (Figure 2A, lanes 8-12). Competition with an excess (200-fold) of unlabeled probe led to disappearance of the TNFα-induced bound complex (Figure 2A, lanes 7 and 14), which confirmed the binding specificity of this assay. Pretreatment for 6 hours with increasing concentrations of pristimerin abrogated TNFα-induced NF-κB-DNA complex formation in a dose-dependent manner (Figure 2B).

To address whether pristimerin exerted a direct inhibitory effect on the binding of NF-κB to DNA, nuclear extracts prepared from untreated KBM5 cells or cells stimulated with TNFα were incubated in a cell-free reaction system in the presence of increasing concentrations of pristimerin. Pristimerin, even at a concentration as high as 20 μM, did not block the TNFα-induced formation of NF-κB-DNA complex (Figure 2C), suggesting that pristimerin did not directly interfere with binding of NF-κB to DNA in a purified nuclear extract.

### Pristimerin inhibits TNFα-induced degradation of IκBα and translocation of p65

**Figure 3:**
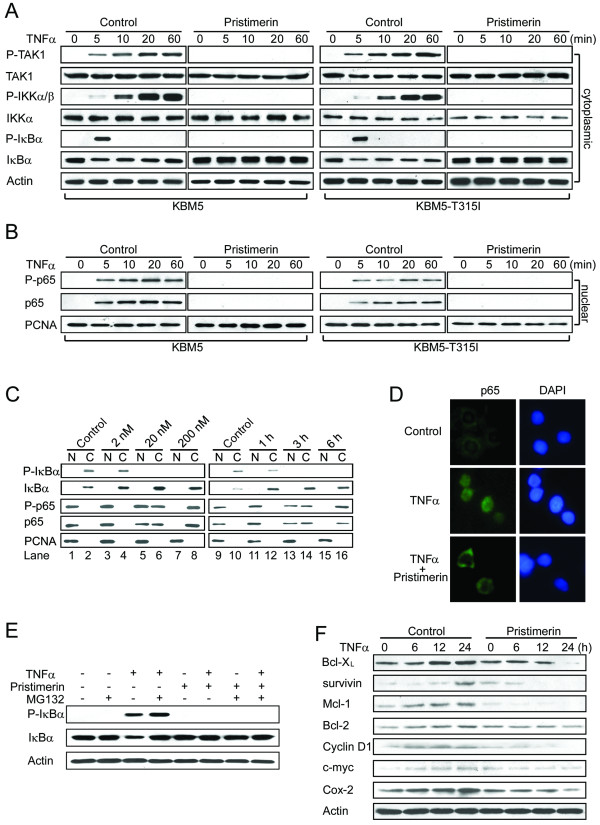
**Pristimerin inhibits TNFα-induced degradation of IκBα and translocation of p65**. KBM5 or KBM5-T315I cells were pretreated with or without 200 nM pristimerin for 6 hours, then treated with TNFα (0.1 nM) at the indicated times; cytoplasmic (A) and nuclear (B) extracts were examined by Western blot analysis with specific antibodies against total and phosphorylated IκBα, IKK and p65, respectively. The same membranes were stripped and reprobed with actin or PCNA. (C) Dose- and time-dependent effect of pristimerin. KBM5 cells were preincubated with the indicated concentrations of pristimerin for 6 hours (left) or 200 nM pristimerin for various durations (right); then treated with TNFα (1 nM) for 5 minutes; cytoplasmic and nuclear extracts were examined by Western blot analysis. (D) Immunofluorescence staining analysis of p65. KBM5 cells were preincubated with 200 nM pristimerin for 6 hours, and TNFα (1 nM) for 5 minutes, fixed in 3% paraformaldehyde, then underwent immunofluorescence analysis against p65 and FITC-conjugated secondary antibody. Nuclei were stained with 4,6-diamidino-2-phenylindole (DAPI). (E) Pristimerin prevented the phosphorylation of IκBα in the presence of proteosome inhibitor. KBM5 cells were treated with 500 nM pristimerin in the absence or presence of MG-132 (0.5 μM) for 6 hours, then treated with TNFα (0.1 nM) for 30 minutes. Cytoplasmic extracts of cells underwent immunoblotting with phosphopecific anti-IκBα. (F) Pristimerin diminishes the expression of NF-κB-regulated proteins involved in survival. Western blot analysis of K562 cells pretreated with 400 nM pristimerin for 6 hours, then stimulated with TNFα (1 nM) for different times.

The findings that pristimerin inhibited TNFα-induced NF-κB transcriptional function and formation of an NF-κB-DNA binding complex predicted that pristimerin would affect IκBα phosphorylation and p65 translocation. To test this hypothesis, KBM5 or KBM5-T315I were pretreated with 200 nM pristimerin for 6 hours, then stimulated with TNFα; cytoplasmic and nuclear extracts were examined by Western blot analysis with antibodies against total and phosphorylated forms of TAK1, IKKα/β, IκBα and p65, respectively. In the absence of pristimerin (Figure 3A, left, control), the levels of phosphorylated TAK1 and phosphorylated IKKα/β in the cytoplasmic fraction of KBM5 cells were appreciably increased starting at 5 minutes after TNFα stimulation as compared with untreated cells. The levels of phosphorylated IκBα appeared as a pulse signal that peaked at 5 minutes after TNFα stimulation and then decreased back to baseline (Figure 3A) as previously reported by Anand et al. [30]. Subsequently (starting 5 minutes after TNFα stimulation), the levels of total IκBα protein in the cytoplasmic fractions decreased, which was consistent with activation of ubiquitination and proteasomal degradation triggered by the phosphorylation of IκBα [31]. In contrast, pristimerin completely abolished the phosphorylation of IKKα/β and IκBα induced by TNFα (Figure 3A, left, pristimerin). Accordingly, the TNFα-induced IκBα degradation was abrogated by pristimerin. The levels of phosphorylated and total p65 in the nuclear fractions were increased in TNFα-stimulated cells (Figure 3B, left, control). The increase in nuclear p65 was markedly abrogated by the pristimerin (Figure 3B, left, pristimerin). Similar findings were obtained in KBM5-T315I cells (Figure 3A and 3B, right panels). Further, pristimerin time- and dose-dependently abrogated the TNFα-induced IκBα degradation and p65 translocation (Figure 3C). The inhibitory effect of pristimerin on TNFα-induced translocation of p65 was further confirmed by immunofluorescent microscopy (Figure 3D).

### Pristimerin inhibits TNFα-dependent IκBα phosphorylation

Because IαBκ phosphorylation is a critical step to tag the protein and trigger ubiquitination-proteosome-dependent degradation of IκBα [32], we next examined whether pristimerin inhibited the degradation of IκBα via inhibiting IκBα phosphorylation. TNFα stimulation elevated the levels of phosphorylated IκBα in KBM5 cells (Figure 3E), which was potentiated by the presence of the proteosome inhibitor MG-132. However, pristimerin completely blocked the TNFα-induced phosphorylation of IκBα even in the presence of MG-132.

### Pristimerin prevents TNFα from inducing NF-κB-dependent gene products involved in survival and apoptosis

It is believed that the pro-survival function of NF-κB is mediated through NF-κB-regulated expression of cell survival proteins whose genes containing an NF-κB binding site in their promoters (e.g., Bcl-X_L_, survivin, Mcl-1, cyclin D1, c-myc, Bcl-2, and Cox 2) [30]. Because pristimerin inhibits TNFα-induced activation of NF-κB, we hypothesized that it would also inhibit TNFα-induced expression of cell survival proteins. K562 cells were treated with or without 400 nM pristimerin for 6 hours, followed by stimulation with TNFα for up to 24 hours. The levels of Bcl-X_L_, survivin, Mcl-1, cyclin D1, c-myc, Bcl-2, and Cox 2 as detected by immunoblotting were increased after TNFα stimulation (Figure 3F), and the TNFα-induced upregulation of these genes was abolished by pristimerin (Figure 3F).

### Pristimerin decreases Bcr-Abl mRNA and inactivates its downstream signaling

**Figure 4:**
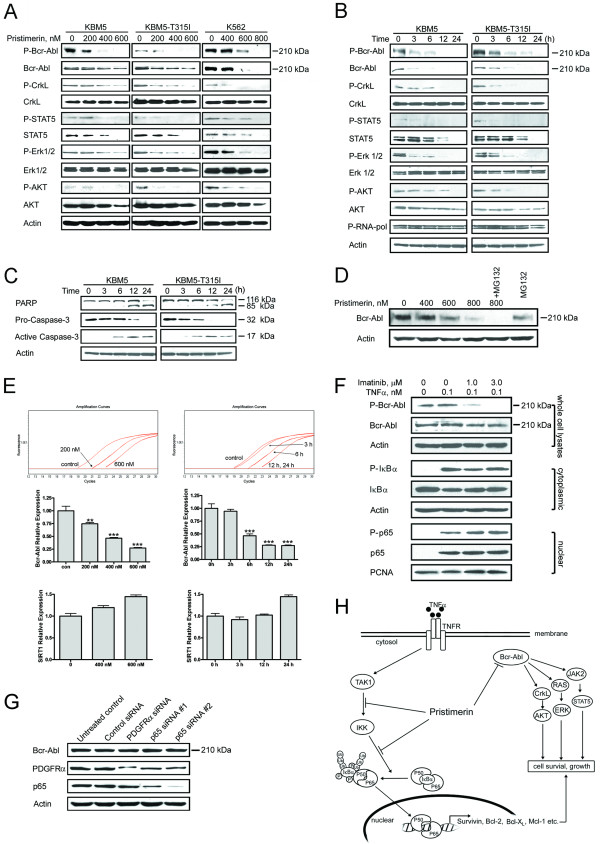
**Decreasing Bcr-Abl and its downstream signaling, and inhibiting NF-κB may be independent mechanisms of actions of pristimerin**. (A) Dose-dependent downregulation of Bcr-Abl protein by pristimerin. CML cells were exposed to pristimerin for 24 hours, whole cell lysates were subjected to immunoblotting analysis. (B and C) CML cells were treated with 600 nM pristimerin for different durations, downstream signaling molecules of Bcr-Abl (B) and proteins of apoptosis (C) were analyzed by Western blotting. (D) K562 cells were pretreated with MG-132 (0.5 μM) for 2 hours, then exposed to pristimerin for another 24 hours. Immunoblots were shown. (E) Pristimerin inhibits Bcr-Abl mRNA levels as measured by RT-qPCR. KBM5 cells were exposed to escalating concentrations of pristimerin for 24 hours (left top and middle) or 600 nM for various durations (right top and middle). The Bcr-Abl mRNA expression relative to the control was calculated by dividing the comparative expression levels. Colomns, mean;bars, 95% confidence intervals, n = 3. Similar data for Sirt1 (an unrelated control gene) were also shown (bottom). **, *P *< 0.01; ***, *P *< 0.0001, one-way ANOVA, post-hoc comparisons, Tukey's test. (F) KBM5 cells were pretreated with 1.0-3.0 μM imatinib for 1 hour, the cells were then stimulated with TNFα (0.1 nM, 5 minutes), NF-κB activation was analyzed with Western blotting. (G) K562 cells were transfected with siRNA duplexes against either human p65, PDGFRα or control siRNA, respectively. Twenty-four hours later, the cells were analyzed by Western blotting. (H) A proposed model to delineate the actions of pristimerin.

We also examined the effect of pristimerin on the expression of Bcr-Abl in CML cells by immunoblotting. Twenty-four-hour treatment with pristimerin dose- and time-dependently decreased levels of Bcr-Abl protein in KBM5, KBM5-T315I or K562 cells (Figure 4A). Bcr-Abl can activate multiple signal transduction pathways (e.g., CrkL, STAT5, Erk1/2, and PI3K/AKT) which can promote cell proliferation and resistance to apoptosis [3]. We reasoned that the loss of total Bcr-Abl protein would lead to a decrease in its kinase activity. We measured autophosphorylation by immunoblotting with the phospho-specific antibody against the phosphorylated Y245 c-Abl as described previously [33]. Basal phosphorylation of Bcr-Abl was detectable in KBM5, KBM5-T315I and K562 cells (Figure 4A). As expected, pritstimerin potently decreased the amount of phosphorylated Bcr-Abl in accord with decrease in the total Bcr-Abl protein (Figure 4A). To better define the effect of pristimerin on Bcr-Abl signaling, we followed the phosphorylation of Bcr-Abl and its downstream targets over time. The levels of Bcr-Abl and phosphorylated Bcr-Abl started to decrease as early as 3 hours after pristimerin treatment, and continued to decrease up to 24 hours after pristimerin treatment (Figure 4B). Concomitantly, the phosphorylation of CrkL, STAT5, Erk1/2 and AKT decreased (Figure 4B). The total protein levels of STAT5 and AKT did decrease at later time points. Taken together, pristimerin decreased Bcr-Abl and inhibited its downstream signaling. Notably, the decrease in Bcr-Abl protein levels preceded the onset of apoptosis, as indicated by specific PARP cleavage and caspase-3 activation after pristimerin treatment (Figure 4C).

To investigate whether the proteosome pathway was involved in the pristimerin-mediated downregulation of Bcr-Abl, we pretreated K562 cells with a sub-cytotoxic concentration (0.5 μM) of the proteosome inhibitor MG-132, but found no effect on the decrease in Bcr-Abl protein levels (Figure 4D). Similarly, the pristimerin-induced decrease of Bcr-Abl protein was not rescued by the presence of caspase-3 inhibitor z-DEVD-fmk (data not shown).

We next examined whether pristimerin inhibited Bcr-Abl at the transcriptional levels. KBM5 and KBM5-T315I cells were exposed to various concentrations of pristimerin for 24 hours (left) or 600 nM for different durations (right); reverse transcription quantitative real-time PCR (RT-qPCR) revealed that the mRNA levels of Bcr-Abl were decreased after treatment with pristimerin (Figure 4E) while the mRNA levels of Sirt1 gene (an unrelated gene control) were not decreased, suggesting a selective activity of pristimerin against Bcr-Abl transcription.

The possibility of interdependence of the two actions of pristimerin (i.e., NF-κB inactivation and depletion of Bcr-Abl) was tested. First, we examined whether the TNFα-induced NF-κB activation was dependent on Bcr-Abl. We pretreated KBM5 cells with 1.0-3.0 μM imatinib for 1 hour to inactivate Bcr-Abl, the cells were then stimulated with TNFα (0.1 nM, 5 minutes), followed by analysis of NF-κB activation. Imatinib treatment led to dephosphorylation of Bcr-Abl, which, however, did not abrogate the TNFα-induced IκBα phosphorylation, and subsequent relocation of p65 (Figure 4F). Second, we employed the siRNA approach to determine the effect of p65 on Bcr-Abl in CML cells. The levels of p65 were appreciably decreased in K562 cells 24 hours after transfection with siRNA duplexes specifically against p65 when compared with control siRNA and PDGFRα siRNA (unrelated gene control) (Figure 4G). Silencing p65 did not affect the levels of Bcr-Abl. The above results together implicate that NF-κB inactivation and Bcr-Abl inhibition may be parallel independent pathways.

### Pristimerin inhibits growth of imatinib-sensitive and imatinib-resistant CML cells

**Figure 5:**
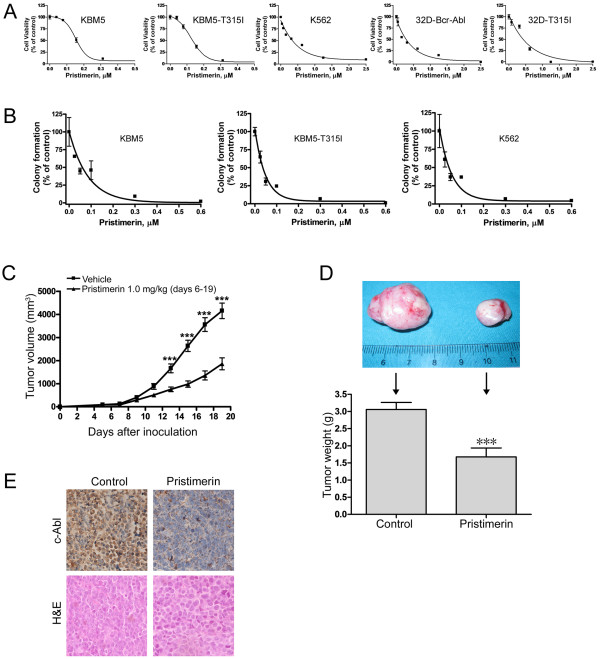
**Pristimerin inhibits growth of imatinib-resistant CML cells in vitro and in vivo**. (A) The indicated 5 lines of CML cells were exposed to escalating concentrations of pristimerin for 72 hours, cell viability was measured by MTS assay. (B) Clonogenicity of CML cells in soft agar was inhibited by pristimerin in a dose-dependent manner. Error bars represent SE. (C, D, E) Pristimerin inhibited the growth of xenografted imatinib-resistant CML cells in a nude mouse model. (C) The growth curves of subcutaneous xenografts of KBM5-T315I cells. Nude mice bearing KBM5-T315I xenograft tumors received intratumoral injection with either 50 μL vehicle [30% Cremophor EL/ethanol (4:1), 70% PBS] or 1.0 mg/kg pristimerin in vehicle daily during days 6-19 after inoculation of KBM5-T315I cells. The estimated tumor volume is plotted versus time (C). Points, mean; Error bars, SE. (D) Weights of tumors dissected on day 19 post-inoculation. Columns, mean; error bars, SE. *** indicates *P *< 0.0001, Student's *t* test. Representative tumors removed from mice of each group. (E) Immnunohistochemical analysis with anti-c-Abl and H & E staining of xenograft tissues from mice sacrificed 19 days after tumor inoculation.

We then evaluated the effect of pristimerin on the growth of CML cells. Human CML cell lines (KBM5, KBM5-T315I and K562), and a pair of murine myeloid cells 32D stably transfected with either the wild-type or T315I human Bcr-Abl were exposed to escalating concentrations of pristimerin for 72 h, followed by the MTS assay. Cell viability of all 5 lines of CML cells was inhibited, with IC_50 _values of 199 nM, 135 nM, 450 nM, 242 nM and 387 nM, respectively (Figure 5A). These data suggest similar sensitivities of imatinib-sensitive and imatinib-resistant CML cells to pristimerin.

Because clonogenicity is believed to better reflect malignant behavior of tumor cells, we compared the impact of pristimerin on clonogenicity in CML cells with that of imatinib. We examined the clonogenicity of KBM5 and KBM-T315I cells after a 24 hour-treatment of imatinib. The IC_50 _values of KBM5 and KBM5-T315I to imatinib were 0.45 μM and 4.10 μM, respectively (data for dose-response curves not shown). The results confirmed the resistance of KBM5-T315I cells to imatinib. In contrast, pristimerin treatment potently inhibited the number of surviving clonogenic KBM5, KBM5-T315I and K562 cells in a dose-dependent manner, with IC_50 _values of 43.7 nM, 35.9 nM and 36.5 nM, respectively (Figure 5B).

### Primary leukemia cells from imatinib-resistant CML patient are also sensitive to pristimerin

We next ascertained the activity of pristimerin against imatinib-resistant primary CML cells. Mononuclear cells in peripheral blood from 1 CML patient who acquired clinical resistance to imatinib versus 4 CML patients that responded to imatinib were exposed to various concentrations of pristimerin for 72 hours. The primary CML cells were sensitive to pristimerin as well, with IC_50_values 6-578 nM (Table 1). In particular, the primary leukemia cells from 1 CML patient with acquired clinical resistance to imatinib were also sensitive to pristimerin (IC_50_value 578 nM). Of interest, primary leukemia cells from 1 patient with JMML (juvenile myelomonocytic leukemia) and 1 patient from ALL (acute lymphoid leukemia) were also sensitive to pristimerin (Table 1).

**Table 1:** IC_50 _values of primary leukemia cells from patients with leukemia to pristimerin.

**Patient no**.	Diagnosis	Imatinib sensitive	Imatinib resistance	IC_50 _(nM)
1	CML		×	578
2	CML	×		6.28
3	CML	×		326
4	CML	×		186
5	CML	×		210
6	JMML			326
7	ALL			205

Note: CML, chronic myelogeneous leukemia; JMML, juvenile myelomonocytic leukemia; ALL, acute lymphoid leukemia.

We also examined the effect of pristimerin on normal bone marrow cells. Normal bone marrow cells collected from 4 healthy donors were treated with increasing concentrations of pristimerin for 72 h; cell viability assayed by MTS indicated that pristimerin was cytotoxic to normal bone marrow cells with a median IC_50 _value of 740.5 nM (range, 681-999 nM). These data were in line with the report by Costa et al. [19] who showed that pristimerin inhibited the cell viability of peripheral blood mononuclear cells with an IC_50 _value of 880 nM. Therefore, bone marrow suppression may be a significant side effect of pristimerin.

### Pristimerin inhibits the growth of xenografted KBM5-T315I cells in nude mice

We next evaluated the *in vivo *activity of pristimerin against imatinib-resistant CML cells using the nude mouse xenograft model. Twenty six *nu/nu *BALB/c mice were injected with KBM5-T315I cells. Five days after inoculation of tumor cells, when the size of tumor reached approximately 50 mm^3^, mice inoculated with KBM5-T315I cells were randomized to receive intratumoral injection with either 50 μL vehicle [30% Cremophor EL/ethanol (4:1), 70% PBS] or 1.0 mg/kg pristimerin in vehicle daily during days 6-19 after inoculation of KBM5-T315I cells. The growth curves (the estimated tumor size calculated from the tumor dimension versus time) are shown in Figure 5C. Pristimerin potently inhibited the growth of KBM5-T315I tumors. The weight of tumors was significantly lower in the treated group than in the control (vehicle) group (Figure 5D, *P *< 0.0001). Immnunohistochemical analysis with an anti-c-Abl antibody (to detect both c-Abl and Bcr-Abl) revealed that c-Abl immunoreactivity was markedly decreased by pristimerin treatment (Figure 5E). These data again pointed to the antitumor activity of pristimerin.

### Pristimerin does not affect cell cycling

**Figure 6:**
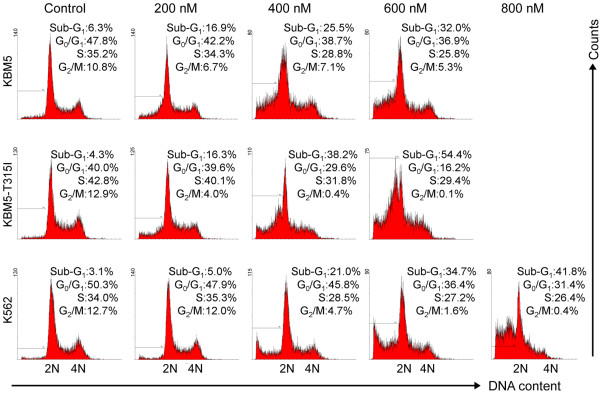
**Effect of pristimerin on cell cycling in CML cells**. KBM5, KBM5-T315I or K562 cells were treated with pristimerin at the indicated concentrations for 24 hours, cell cycling was analyzed by flow cytometry after staining with propidium iodide. Graphs show data from a representative experiment.

After exposing CML cells to various concentrations of pristimerin for 24 hours, cell cycle analysis was conducted by using flow cytometry with propidium iodide staining. The results revealed no significant cell cycle alteration in CML cells treated with various concentrations of pristimerin for 24 hours except for the appearance of the sub-G1 apoptotic population (Figure 6).

### Pristimerin induced apoptosis in imatinib-sensitive and imatinib-resistant CML cells

**Figure 7:**
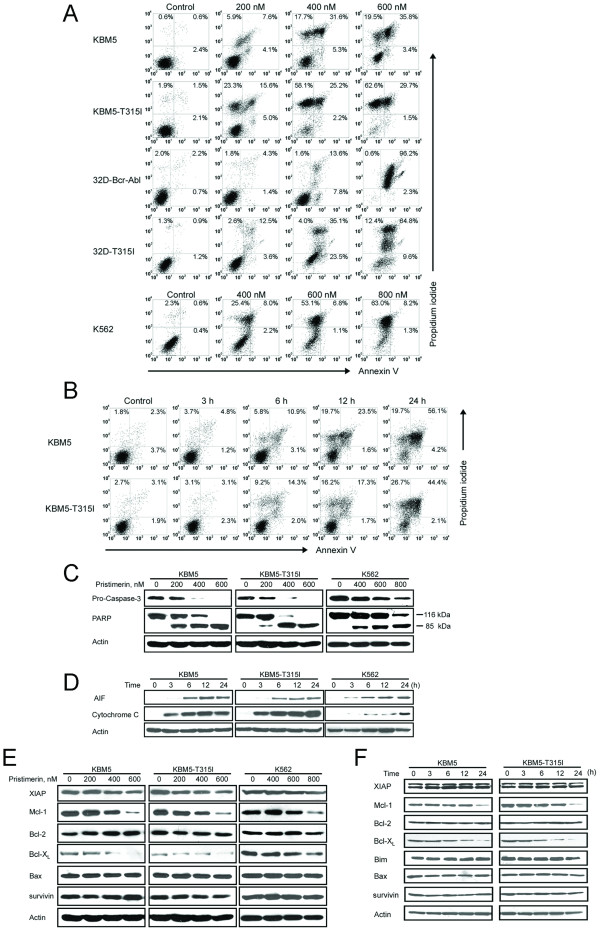
**Pristimerin induces apoptosis in imatinib-sensitive and -resistant CML cells by eliciting mitochondrial damage**. (A, B, C) CML cells were exposed to pristimerin with concentrations ranging from 0 to 600 nM for 24 hours (A), or 600 nM pristimerin for various durations (B), then the cells underwent Annexin V/PI double staining for apoptotic cell death assay. Parallel samples were lysed for immunoblotting analysis with antibody against caspase-3 and PARP (C). (D) Pristimerin induced mitochondrial release of cytochrome c and apoptosis-inducing factor (AIF). KBM5, KBM5-T315I or K562 cells were treated with pristimerin at 600 nM, 600 nM and 800 nM, respectively, for 0-24 hours; levels of cytochrome c in the cytosolic extracts prepared using a digitonin buffer were detected by Western blot analysis. (E) Western blot analysis of apoptosis-related proteins such as Bcl-2, Bcl-X_L_, Mcl-1 and survivin was shown. The indicated CML cells were exposed to pristimerin for 24 h. (F) Immunoblotting analysis of apoptosis-related proteins in response to pristimerin (600 nM) for different durations was shown.

The ability for pristimerin to induce apoptosis in CML cells was assessed by flow cytometry after staining with Annexin V and propidium iodide. After treatment with pristimerin apoptosis was induced in a dose- and time-dependent manner (Figure 7A, 7B) in the CML cells. In parallel, Western blotting analysis revealed that pristimerin induced a dose-dependent specific cleavage of PARP, which is a hallmark of apoptosis (Figure 7C). Concomitantly, pristimerin led to a decline of the precursor form of caspase-3, reflecting its activation.

Most chemotherapeutic agents induced apoptosis by triggering release of cytochrome c and AIF from mitochondria into the cytosol. To evaluate the apoptosis pathway activated by pristimerin, CML cells were exposed to pristimerin, cytochrome c and AIF in the cytosolic fraction was examined by Western blotting at different time points. Cytochrome c and AIF were undetectable in the cytosol of control cells, but were released from the mitochondria into the cytosol after pristimerin treatment (Figure 7D). The results implied that pristimerin triggered the mitochondrial pathway of apoptosis. Pristimerin activated the same apoptotic pathway in breast cancer cells as reported by Wu C et al. [21] The effect of pristimerin on antiapoptotic proteins (Bcl-2, Bcl-X_L_, Mcl-1, survivin) of the Bcl-2 gene family regulating the mitochondrial pathway of apoptosis was also examined. Pristimerin treatment decreased the levels of Bcl-X_L _and Mcl-1 in a dose- and time-dependent manner without affecting Bcl-2, XIAP, Bax and survivin (Figure 7E and 7F).

## Discussion

The prognosis of CML resistant to imatinib is poor [8]. Bcr-Abl-T315I mutation is a major cause to confer resistance to STI571 as well as the second generation of tyrosine kinase inhibitors. NF-κB may represent an important mechanism to prevent apoptosis in tumor cells. In the present study, we discovered that pristimerin potently blocked NF-κB signaling and dramatically downregulated Bcr-Abl mRNA regardless of the mutational status of Bcr-Abl, inhibited growth and induced apoptosis in CML cells harboring wild-type Bcr-Abl or Bcr-Abl-T315I mutation. We confirmed this activity with two pairs of CML cell lines (KBM5 versus KBM5-T315I, 32D-Bcr-Abl versus 32D-Bcr-Abl-T315I) and primary cells from CML patients. Additionally, pristimerin inhibited the growth of imatinib-resistant Bcr-Abl-T315I in nude mouse xenografts. To our knowledge, this is the first report to show that pristimerin is effective *in vitro *and *in vivo *against CML cells, including those with the T315I mutation. Because none of the currently available tyrosine kinase inhibitors in clinical use are effective against CML cells bearing T315I-Bcr-Abl, our findings are of particular interest.

Inhibition of proteasomes was reported as a mechanism by which pristimerin induces apoptosis in prostate cancer cells [34]. However, inhibition of proteasome occurs at micromolar concentrations (pristimerin at 2.2-3.0 μM inhibited 50% of the proteolytic activity of purified 20S proteasome; pristimerin at 5.0 μM inhibited proteasomal activity by 30%-40% in tumor cells [34]) while the inhibition of Bcr-Abl transcription and inactivation of NF-κB signaling occur in nanomolar concentrations (Figures 3 and 4). Moreover, no increased cell death in CML cells was observed when pristimerin was combined with MG-132 (a proteasome inhibitor). Therefore, inhibition of proteasomes is unlikely to be a relevant mechanism by which pristimerin induces apoptosis in CML cells.

The inhibitory action of pristimerin against NF-κB has been documented in murine macrophages (RAW 264.7) induced by lipopolysaccharide [18] and in human myeloma cells induced by TNFα [20]. In these previous reports, only NF-κB-dependent reporter gene assay and EMSA data were reported; the precise steps in NF-κB signaling that were inhibited by pristimerin were not ascertained. In this study, we thoroughly examined the effect of pristimerin on NF-κB signaling beyond NF-κB-dependent gene reporter and DNA binding of NF-κB (EMSA) to include IκBα phosphorylation, translocation of p65, and expression of NF-κB-regulated genes. We pinpointed the precise steps at which pristimerin exerted its inhibitory action in the signaling cascade of NF-κB: TAK1→IKK and IKK→IκBα (Figure 3A; Model in Figure 4H).

Oncogenic mutations such as Bcr-Abl, PDGFRα, KIT, and Ras have been demonstrated to lead to constitutive activation of NF-κB [16], [35-38]. Reuther et al. demonstrated that NF-κB activation was essential for Bcr-Abl-induced transformation [37]. Inactivation of Bcr-Abl, Flt3 and PDGFRα by specific inhibitors can ablate the NF-κB activation [12]. By this potential mechanism, rapid decrease of Bcr-Abl by pristimerin may cause or enhance inactivation of NF-κB signaling. Despite the fact that Bcr-Abl kinase is sufficient to elicit constitutive activation of NF-κB, NF-κB activation may also occur via an oncogenic mutations-independent mechanism. For instance, inhibition of Flt3 by its inhibitor AG1296 in primary AML blast cells variably affected constitutive NF-κB activation, indicating that a Flt3-independent mechanism of NF-κB activation existed [35,39]. Additionally, cytokines including TNFα are increased in the serum of patients with CML compared with healthy individuals [40]. The abnormal microenvironment in CML bone marrow likely causes deregulated secretion of cytokines by stromal cells [41]. Of note, our data revealed that inactivation of Bcr-Abl by imatinib did not interfere with the TNFα-induced NF-κB activation as reflected by IκBα phosphorylation and subsequent relocation of p65. Moreover, silencing p65 did not affect the levels of Bcr-Abl. Together, our results implicate that NF-κB inactivation and Bcr-Abl inhibition may be parallel pathways for pristimerin actions. Future work should assess the relative importance of these two mechanisms.

Pristimerin decreased mRNA of Bcr-Abl. Our data did not support that pristimerin is a global transcription inhibitor (e.g. triptolide [42] and flavopiridol [33]), because pristimerin treatment did not affect the activity of RNA polymerase (Figure 4B) and pristimerin did not inhibit the mRNA of Sirt1 (an unrelated gene, Figure 4E). These results suggest a relatively specific activity of pristimerin against Bcr-Abl mRNA. Further research will be required to identify the precise mechanism by which pristimerin decreases Bcr-Abl mRNA.

CML, being highly dependent on presence of Bcr-Abl, is one of the typical models of oncogene addiction [43]. Inactivation of Bcr-Abl kinase by tyrosine kinase inhibitors (e.g. imatinib, dasatinib) or downregulating the total levels of cellular Bcr-Abl by pristimerin, triptolide [22] and flavopiridol [33] (at the mRNA level), 17-AAG [44] (at the protein level) could be effective approaches to block "addictive oncogene" in Bcr-Abl-expressing cells. Although pristimerin, triptolide, flavopiridol and 17-AAG may impact multiple molecules, Bcr-Abl downregultion is the common effect and is likely the major factor inducing apoptosis in Bcr-Abl-expressing cells. The decrease of Bcr-Abl preceded the onset of apoptosis, suggesting that Bcr-Abl may be the cause and not a consequence of apoptosis in CML cells. Nevertheless, pristimerin is effective in killing imatinib-resistant CML cells bearing T315I Bcr-Abl. Furthermore, pristimerin may be effective against CML cells bearing the Bcr-Abl with mutations other than T315I because of its effect on Bcr-Abl mRNA, and this remains to be confirmed. It also remains to be investigated whether pristimerin has activity against CML stem cells, which is believed to be a critical cause of imatinib-resistance and an obstacle to curing CML.

Pristimerin has been tested *in vivo *by us (Figure 5) and others [20]. Suppression of tumor growth in mice treated with pristimerin without causing death of the mice is proof that a therapeutic window exists. The cytotoxicity of pritimerin against normal bone marrow cells suggests that bone marrow suppression may be a potential side effect of pristimerin. The translational potential of pristimerin to clinical use will depend on its toxicity profile in Phase I trials. At the very least, pritimerin is a lead compound for further development of triterpenoid-derived anticancer agents. In the future, it will be interesting to examine whether bone marrow protective agents are helpful in counteracting this side effect of pristimerin. Modifying the structure of pristimerin may be an alternative approach to decrease the toxicity of the compound.

## Conclusions

In summary, our findings together showed that pristimerin had an activity against CML cells bearing wild-type or Bcr-Abl-T315I mutation. The mechanisms might involve inhibition of NF-κB and Bcr-Abl. We concluded that pristimerin could be a lead compound that merited further development to overcome the imatinib-resistance of CML patients.

## Abbreviations

AIF: apoptosis-inducing factor; ALL: acute lymphoid leukemia; AML: acute myelogeneous leukemia; CML: chronic myelogeneous leukemia; EMSA: electrophoretic mobility shift assay; FCS: fetal calf serum; JMML: juvenile myelomonocytic leukemia; NF-κB: nuclear factor κB; PCNA: proliferating cell nuclear antigen; RT-qPCR: reverse transcription quantitative real-time PCR; TNFα: tumor necrosis factor α.

## Competing interests

The authors declare that they have no competing interests.

## Authors' contributions

ZL performed experiments presented in Figures 4, 5, 6, 7; YJ performed experiments presented in Figures 1, 2, 3, 4; CC and JL provided leukemia patient specimens and relevant clinical data, and analyzed the data; QC performed experiments of RT-qPCR in Figure 4E; JP designed, performed research, analyzed data, directed the whole study and wrote the manuscript. All the authors read and proved the final manuscript.

## Supplementary Material

Additional file 1 **Table S1**. Characteristics of patients with leukemia. Summary of clinical characteristics of patients with leukemia. Click here for file
