# Correction to: Pristimerin induces apoptosis in imatinib-resistant chronic myelogenous leukemia cells harboring T315I mutation by blocking NF-κB signaling and depleting Bcr-Abl

**DOI:** 10.1186/s12943-021-01414-7

**Published:** 2021-12-31

**Authors:** Zhongzheng Lu, Yanli Jin, Chun Chen, Juan Li, Qi Cao, Jingxuan Pan

**Affiliations:** 1 Department of Pathophysiology, Zhongshan School of Medicine, Sun Yat-sen University, Guangzhou, PR China; 2 Department of Pediatrics, Sun Yat-sen Memorial Hospital, Sun Yat-sen University, Guangzhou, PR China; 3 Department of Hematology, The First Affiliated Hospital, Sun Yat-sen University, Guangzhou, PR China

**Correction to: Mol Cancer 9, 112 (2010)**

**https://doi.org/10.1186/1476-4598-9-112**

Following publication of the original article [1], minor errors were identified in the images presented in Fig. 3; specifically:

- Fig. 3a: immunoblots for p-TAK1 in KBM5 and KBM5-T315I cells treated with Pristimerin; both images have been replaced with the correct image
- Fig. 3a: immunoblots for p-IKKα/β in KBM5 and KBM5-T315I cells treated with Pristimerin; both images have been replaced with the correct image
- Fig. 3a: immunoblots for p-IκBα in KBM5 and KBM5-T315I cells treated with Pristimerin; both images have been replaced with the correct image
- Fig. 3b: immunoblots for p-p65 in KBM5 and KBM5-T315I cells treated with Pristimerin; both images have been replaced with the correct image
- Fig. 3b: immunoblots for p65 in KBM5 and KBM5-T315I cells treated with Pristimerin; both images have been replaced with the correct image

The corrected figure is given below. The correction does not have any effect on the results or conclusions of the paper. The original article has been corrected.

**Fig. 3:**
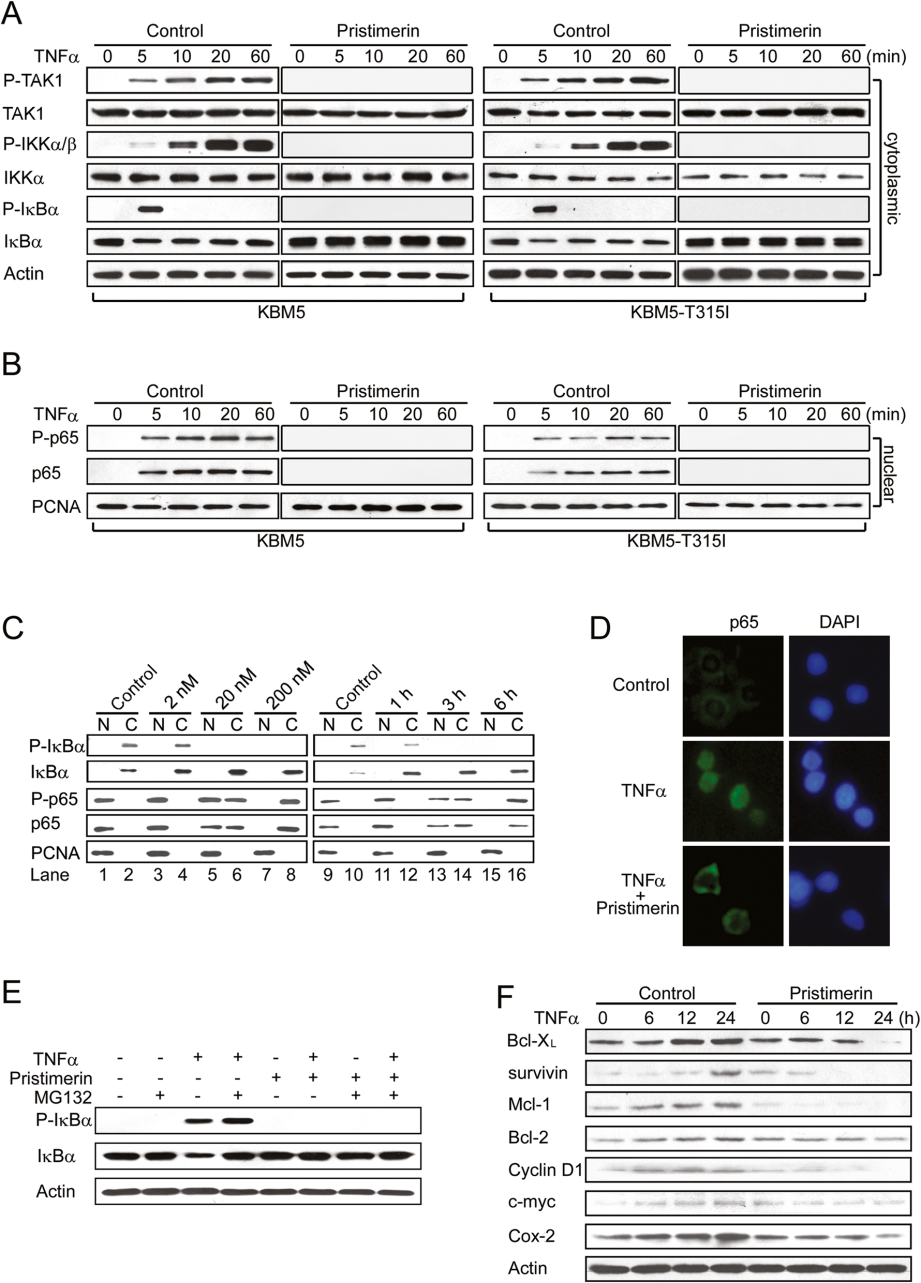
Pristimerin inhibits TNFα-induced degradation of IκBα and translocation of p65. KBM5 or KBM5-T315I cells were pretreated with or without 200 nM pristimerin for 6 hours, then treated with TNFα (0.1 nM) at the indicated times; cytoplasmic (A) and nuclear (B) extracts were examined by Western blot analysis with specific antibodies against total and phosphorylated IκBα, IKK and p65, respectively. The same membranes were stripped and reprobed with actin or PCNA. (C) Dose- and time-dependent effect of pristimerin. KBM5 cells were preincubated with the indicated concentrations of pristimerin for 6 hours (left) or 200 nM pristimerin for various durations (right); then treated with TNFα (1 nM) for 5 minutes; cytoplasmic and nuclear extracts were examined by Western blot analysis. (D) Immunofluorescence staining analysis of p65. KBM5 cells were preincubated with 200 nM pristimerin for 6 hours, and TNFα (1 nM) for 5 minutes, fixed in 3% paraformaldehyde, then underwent immunofluorescence analysis against p65 and FITC-conjugated secondary antibody. Nuclei were stained with 4,6-diamidino-2-phenylindole (DAPI). (E) Pristimerin prevented the phosphorylation of IκBα in the presence of proteosome inhibitor. KBM5 cells were treated with 500 nM pristimerin in the absence or presence of MG-132 (0.5 μM) for 6 hours, then treated with TNFα (0.1 nM) for 30 minutes. Cytoplasmic extracts of cells underwent immunoblotting with phosphopecific anti-IκBα. (F) Pristimerin diminishes the expression of NF-κB-regulated proteins involved in survival. Western blot analysis of K562 cells pretreated with 400 nM pristimerin for 6 hours, then stimulated with TNFα (1 nM) for different times.
