# Supplementary material for: Pristimerin induces apoptosis in imatinib-resistant chronic myelogenous leukemia cells harboring T315I mutation by blocking NF-κB signaling and depleting Bcr-Abl
Source: Mol Cancer. 2010 May 19;9:112. doi: 10.1186/1476-4598-9-112 (PMC2893099; doi:10.1186/1476-4598-9-112)
Supplement: Additional file 1 — Table S1. Characteristics of patients with leukemia. Summary of clinical characteristics of patients with leukemia. [file 1476-4598-9-112-S1.DOC]

Additional file 1

Table S1. Characteristics of patients with leukemia

| Patient No. | Age (yr)/Sex | Diagnosis | Date of diagnosis | Prior Therapy | Date imatinib therapy began | Date resistance recorded | Time to Progression (months) | Stage | Blast% | WBC count (10^9^/L) | Bcr-Abl positive |
| --- | --- | --- | --- | --- | --- | --- | --- | --- | --- | --- | --- |
| 1 | 29/F | CML | 7/2005 | IM, Hu | 7/2005 | 7/2009 | 48 | AP | 11 | 10.1 | Yes |
| 2 | 54/F | CML | 8/2009 | - | - | - | - | CP | 6.5 | 153.4 | Yes |
| 3 | 56/F | CML | 10/2009 | - | - | - | - | CP | ND | 136.6 | Yes |
| 4 | 7/F | CML | 8/2009 | Hu | - | - | - | CP | 3 | 188 | Yes |
| 5 | 13/M | CML | 8/2009 | - | - | - | - | CP | ND | 334 | Yes |
| 6 | 8/M | JMML | 8/2009 | - | - | - | - | AP | 13.6 | 23.6 | No |
| 7 | 23/F | ALL | 6/2009 | - | - | - | - | CP | 18 | 16.2 | ND |

Note: CML, chronic myelogeneous leukemia; JMML, juvenile myelomonocytic leukemia; ALL, acute lymphoid leukemia; AP, accelerated phase; CP, chronic phase; IM, imatinib; Hu, hydroxyurea; ND, not detection.
